# Completed genome and emergence scenario of the multidrug-resistant nosocomial pathogen *Staphylococcus epidermidis* ST215

**DOI:** 10.1186/s12866-024-03367-5

**Published:** 2024-06-19

**Authors:** Therese Kellgren, Chinmay Dwibedi, Micael Widerström, David Sundell, Caroline Öhrman, Andreas Sjödin, Tor Monsen, Patrik Rydén, Anders Johansson

**Affiliations:** 1 Department of Mathematics and Mathematical Statistics, Umeå University, Umeå, SE 90187 Sweden; 2 Department of Clinical Microbiology and Molecular Infection Medicine Sweden (MIMS), Umeå University, 90185 Umeå, Sweden; 3 Department of Clinical Microbiology, Umeå University, 90185 Umeå, Sweden; 4 Division of CBRN Defence and Security, Swedish Defense Research Agency, 90182 Umeå, SE Sweden

**Keywords:** *Staphylococcus epidermidis*, Cross infection/epidemiology, Healthcare-associated infections, Whole-genome sequencing, Drug resistance, multiple, bacterial multidrug resistance

## Abstract

**Background:**

A multidrug-resistant lineage of *Staphylococcus epidermidis* named ST215 is a common cause of prosthetic joint infections and other deep surgical site infections in Northern Europe, but is not present elsewhere. The increasing resistance among *S. epidermidis* strains is a global concern. We used whole-genome sequencing to characterize ST215 from healthcare settings.

**Results:**

We completed the genome of a ST215 isolate from a Swedish hospital using short and long reads, resulting in a circular 2,676,787 bp chromosome and a 2,326 bp plasmid. The new ST215 genome was placed in phylogenetic context using 1,361 finished public *S. epidermidis* reference genomes. We generated 10 additional short-read ST215 genomes and 11 short-read genomes of ST2, which is another common multidrug-resistant lineage at the same hospital. We studied recombination’s role in the evolution of ST2 and ST215, and found multiple recombination events averaging 30–50 kb. By comparing the results of antimicrobial susceptibility testing for 31 antimicrobial drugs with the genome content encoding antimicrobial resistance in the ST215 and ST2 isolates, we found highly similar resistance traits between the isolates, with 22 resistance genes being shared between all the ST215 and ST2 genomes. The ST215 genome contained 29 genes that were historically identified as virulence genes of *S. epidermidis* ST2. We established that in the nucleotide sequence stretches identified as recombination events, virulence genes were overrepresented in ST215, while antibiotic resistance genes were overrepresented in ST2.

**Conclusions:**

This study features the extensive antibiotic resistance and virulence gene content in ST215 genomes. ST215 and ST2 lineages have similarly evolved, acquiring resistance and virulence through genomic recombination. The results highlight the threat of new multidrug-resistant *S. epidermidis* lineages emerging in healthcare settings.

**Supplementary Information:**

The online version contains supplementary material available at 10.1186/s12866-024-03367-5.

## Background

*Staphylococcus epidermidis* is a natural colonizer of human skin and an important opportunistic nosocomial pathogen responsible for many healthcare-associated infections, particularly device- and implant-related infections [1]. Variation in the gene content among strains of *S. epidermidis* contributes to the fact that some strains form biofilms on surgical implants and can resist the effects of antimicrobial drugs that were once effective against this microbe. Strains with genes encoding virulence factors and antimicrobial drug resistance are responsible for most healthcare-associated infections and the increased health-care costs associated with *S. epidermidis* [2, 3]. *S. epidermidis* isolated from healthy humans in the community are more diverse and generally susceptible to several antimicrobial drugs that can be used for treatment [4–7].

Previous work has shown that hospital-associated multidrug-resistant genetic lineages of *S. epidermidis* are widely present in healthcare settings [8–12]. Analyses by multilocus sequence typing (MLST) of *S. epidermidis* revealed that a few nucleic acid sequence types (STs), predominantly ST2 and ST5, are dominant causes of infection worldwide. Numerous additional STs share genetic ancestry with the dominating STs and collectively form a very large clonal complex of *S. epidermidis* that consists of hundreds of related STs found in healthcare settings [13, 14]. Analyses of genetic inheritance patterns in large sets of isolates with MLST data revealed well-supported genetic clusters and that genetic recombination has been important in shaping the *S. epidermidis* population [15]. Among the individual STs, ST2 is the most common and best described, and much of the functional knowledge of putative virulence genes and genes encoding antibiotic resistance was derived from studies of multidrug-resistant ST2 isolates [6, 13, 14, 16, 17]. Recently, ST2 and ST23 isolates with *rpoB* gene mutations were shown to be associated with rifampicin resistance and heteroresistance to glycopeptides, which is problematic since vancomycin is the preferred treatment for most infections caused by *S. epidermidis* [18, 19].

In Northern Europe, a multidrug-resistant ST named ST215 is an emerging cause of healthcare-associated *S. epidermidis* infections [11]. In Sweden, ST215 and ST2 are found at approximately equal frequencies in infected knee and hip prostheses, with a 30/30% distribution among all the different *S. epidermidis* STs causing infections [11, 20, 21]. Recent work using whole-genome sequencing of *S. epidermidis* from Sweden showed that ST215 does not share a recent common ancestor with ST2 or other common STs [22]. Other work using whole-genome sequencing indicated that multidrug-resistant STs have emerged independently from various *S. epidermidis* genetic backgrounds [23, 24].

In this work, we generated a complete genome sequence of the recently emerged multidrug-resistant and nosocomial *S. epidermidis* ST215 strain that appears to be geographically constrained to northern Europe, including Sweden. In addition, we generated additional draft genomes of ST215 isolates from Sweden and ST2 isolates from Sweden and Australia and performed genomic and antimicrobial drug resistance phenotype comparisons of recently emerged ST215 and well-established ST2 strains with a worldwide distribution.

## Results

### Clinical context and antimicrobial susceptibility

The 23 *S. epidermidis* isolates analyzed in detail here were cultured from clinical specimens collected from deep sternal wounds, central line-related sepsis or infected implanted medical devices such as joint prostheses. Diagnostic culturing has been performed in clinical microbiology laboratories serving healthcare in Perth, Australia; in Umeå, Sweden; or in Östersund, Sweden (Supplementary Table S1).

**Fig. 1:**
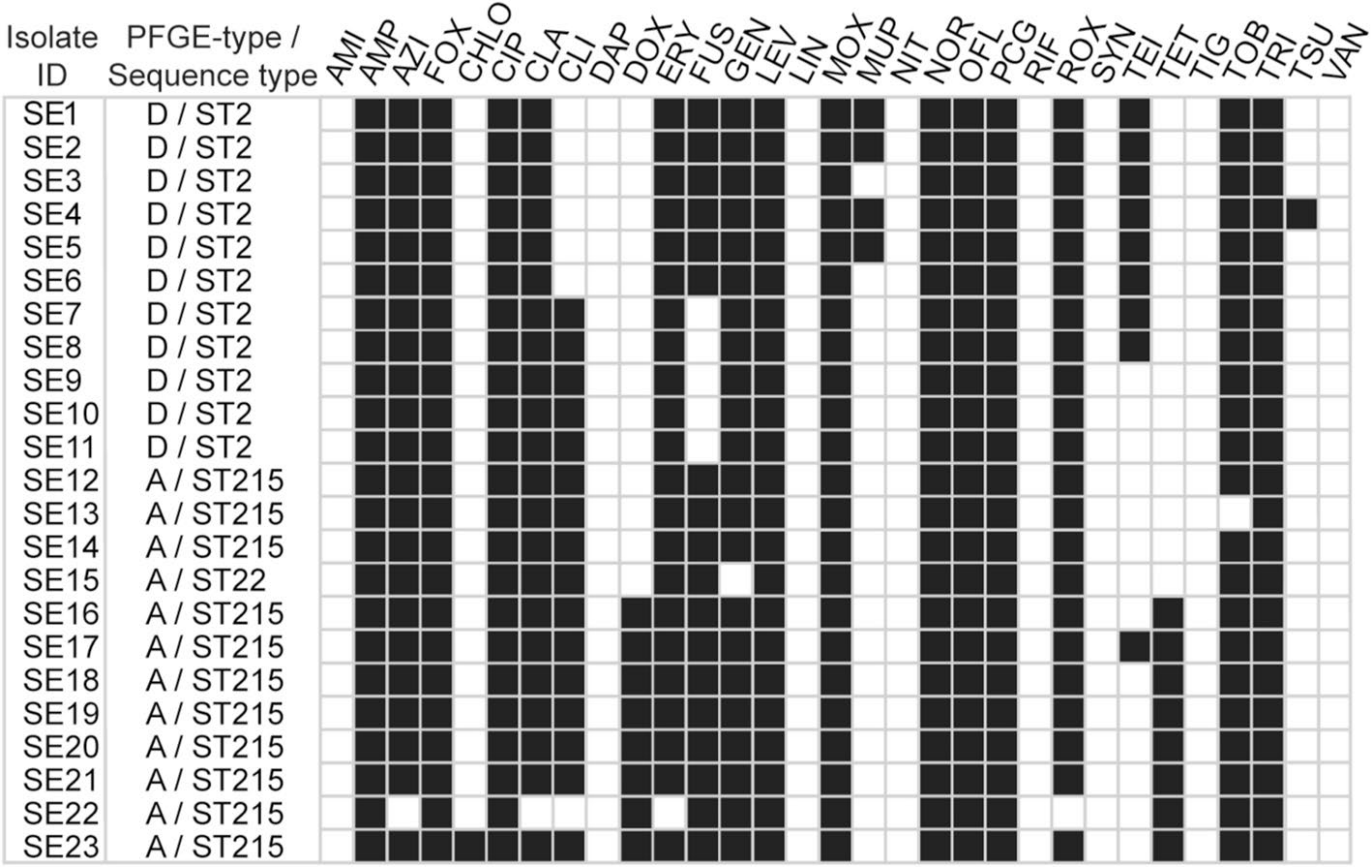
Antimicrobial susceptibilities of 23 *S. epidermidis* isolates toward 31 antimicrobial agents. Black indicates resistance, and white indicates susceptibility according to EUCAST breakpoints. Abbreviations: AMI, Amikacin; AMP, Ampicillin; AZI, Azithromycin; AZT, Aztreonam; CHLO, Chloramphenicol; CIP, Ciprofloxacin; CLA, Clarithromycin; CLI, Clindamycin; DAP, Daptomycin (+ 50 mg/L Ca2 +); DOX, Doxycycline; ERY, Erythromycin; FOX, Cefoxitin; FUS, Fusidic acid; GEN, Gentamicin; LEV, Levofloxacin; LIN, Linezolid; MOX, Moxifloxacin; MUP, Mupirocin; MUP-high, Mupirocin-high concentration; NIT, Nitrofurantoin; NOR, Norfloxacin; OFL, Ofloxacin; PCG, Benzylpenicillin; RIF, Rifampicin; ROX, Roxithromycin; SYN, Quinupristin-dalfopristin; TEI, Teicoplanin; TET, Tetracycline; TIG, Tigecycline; TOB, Tobramycin; TRI, Trimethoprim; TSU, Trimethoprim-sulfamethoxazole; and VAN, Vancomycin

The antimicrobial susceptibility of the 23 *S. epidermidis* isolates was tested using a panel of 31 antimicrobial drugs, and the isolates were found to be resistant to multiple antimicrobial agents, as shown in Fig. 1.

The 23 isolates were generally resistant to cefoxitin and other β-lactam antimicrobial agents and to at least 4 out of 13 additional distinct antimicrobial classes (fusidic acid, mupirocin, macrolides/clindamycin, tetracyclines, quinolones, gentamicin/tobramycin, rifampicin, chloramphenicol, nitrofurantoin, daptomycin, linezolid, folate pathway inhibitors, and glycopeptides). Broth dilution MIC values determined using the EUCAST methodology are detailed in Supplementary Table S2.

### Genome phylogenies

**Fig. 2:**
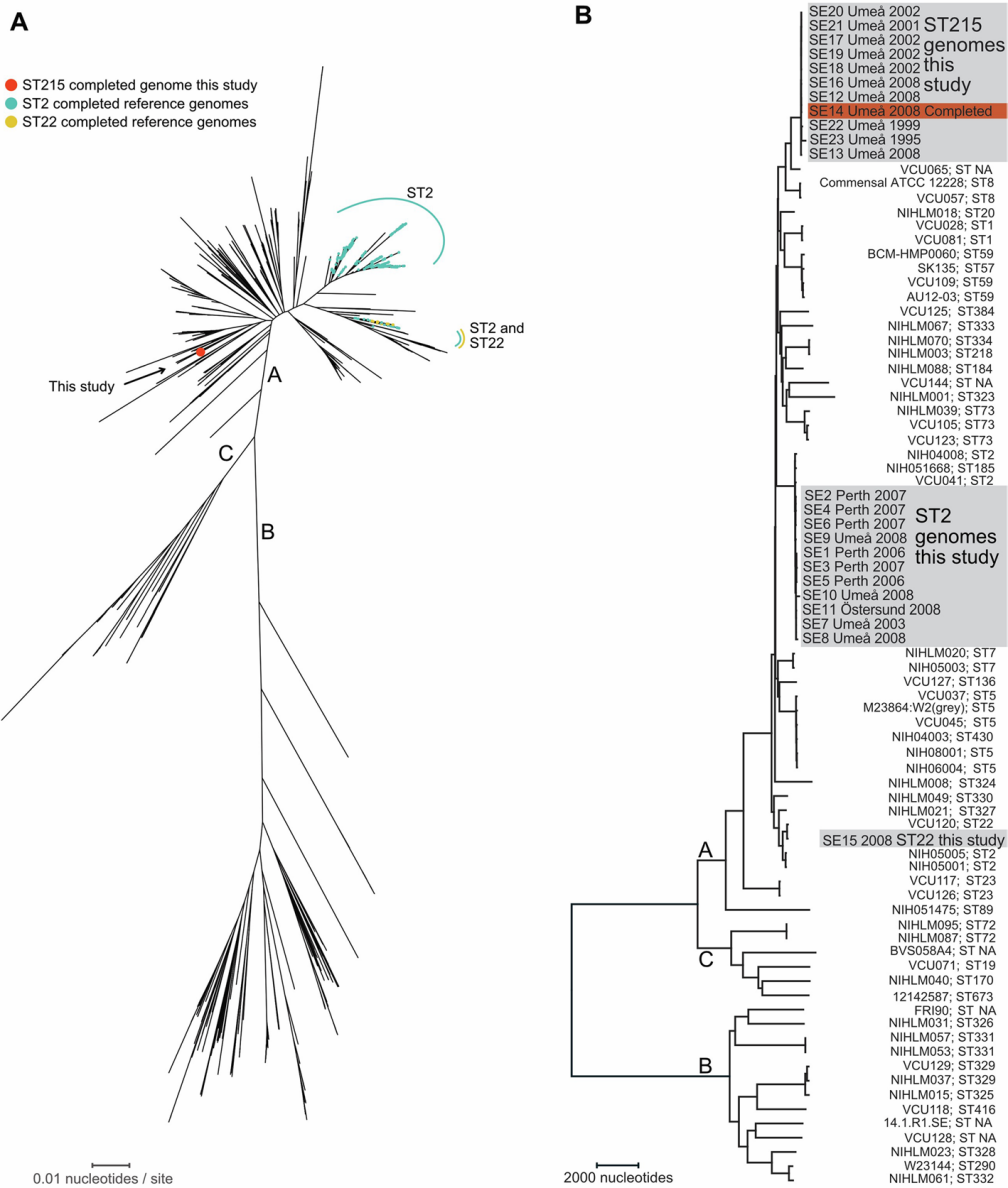
Whole-genome phylogenies representing the known species diversity of *S. epidermidis,* including the isolates sequenced for this study. Panel A is a maximum likelihood phylogeny based on the alignment of 1,361 completed reference genomes and the completed ST215 genome generated for this study (red filled circle) with 2,456 variable sites. Three previously described major genomic clusters of *S. epidermidis,* designated A, B, and C, and the phylogenetic positions of the previously described genetic lineages ST2 and ST22 are indicated. The scale bar indicates the number of nucleotide substitutions per nucleotide site. Panel B shows a neighbor-joining phylogeny based on the mapping of short reads to the index genome ATCC 12228 and includes 23 healthcare-associated *S. epidermidis* genomes generated for this work in the context of 60 diverse *S. epidermidis* genomes. Three previously described major genomic clusters of *S. epidermidis,* designated A, B, and C, are indicated and correspond to the same major clusters in panel A. Strain names and sequence types (STs) are shown on the right. The scale bar indicates the number of nucleotide substitutions along the tree branches

We identified the Illumina short-read sequenced isolates as belonging to ST2 (n = 11), ST215 (n = 11) and ST22 (n = 1) as defined by the *S. epidermidis* MLST scheme [25]. By combining Illumina and Nanopore long-read sequencing data, we finished one ST215 genome (clinical isolate SE14) and identified one 2,676,785 bp circular chromosome and one 2,326 bp circular plasmid. The average depth of the chromosome coverage was 871 × for Illumina and 78 × for Nanopore, and the plasmid coverage was 340 × and 54 × , respectively. We saved all reads in excess not mapping to the two contigs (approximately 1,000 (0.2%) nanopore reads and 200,000 (0.16%) Illumina reads) but found no evidence for additional plasmids by reassembly. We calculated a complete *S. epidermidis* whole-genome phylogeny based on multiple alignments using 1361 publicly available completed genomes representing 58 STs to place ST215 within the total known genomic diversity of the species (Fig. 2A). ST215 clustered with the previously described major genomic cluster A in a phylogeny with two additional major clusters, C and B, reproducing previous findings [23, 24]. In accordance with a prior study, the ST2 genomes exhibited a paraphyletic pattern characterized by two distinct groups of ST2 genomes that did not share a recent common ancestor. To put all 23 isolates sequenced in the context of multiple described healthcare-associated STs, we calculated an additional genome phylogeny based on short-read data with a set of 60 *S. epidermidis* genomes, including STs commonly described from healthcare (Supplementary Table S3) [23]. We mapped the short reads on a 1,613,625-nucleotide core and found 2,456 single-nucleotide polymorphisms, resulting in a tree that reproduced the division of the major clusters A, B, and C and showed that 11 ST215 genomes and 11 ST2 genomes clustered tightly but separately within major cluster A (Fig. 2B).

Hereafter, we analyzed and compared all the ST2 and ST215 genomes generated for this study to explore differences and similarities between the two genetic lineages, which are the two most common multidrug-resistant *S. epidermidis* lineages in hospitals in Sweden.

### Analyses of homologous recombination in ST215 and ST2

**Fig. 3:**
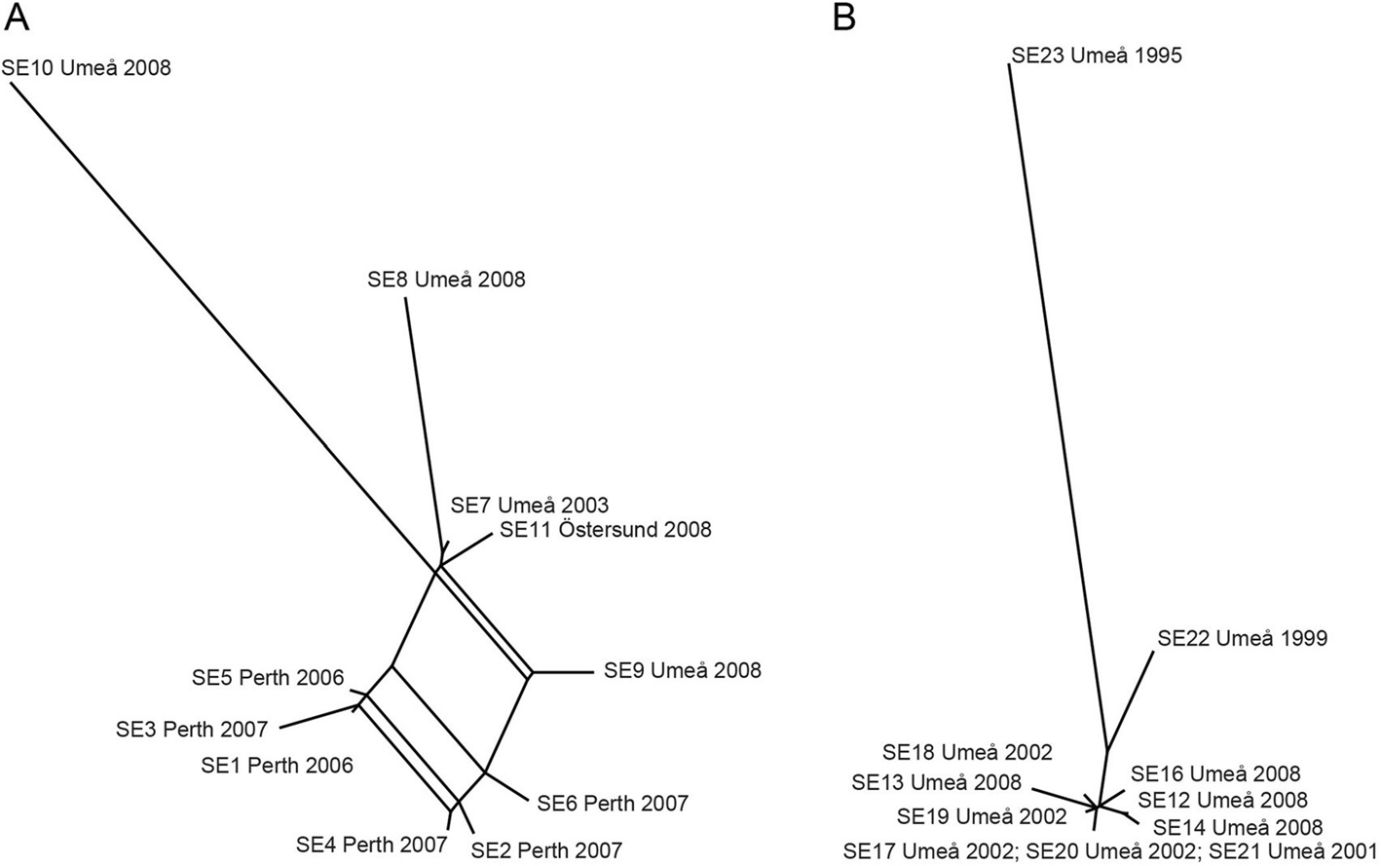
Phylogenetic network graphs with conflicting phylogenetic signals shown as a reticular pattern. Panel **A** shows the ST2 genomes. Panel **B** shows the ST215 genomes

By constructing unrooted phylogenetic networks using Splits Tree3.2 software, we found a greater network among the genomes assigned to ST2 than among the ST215 genomes, suggesting that recombination events caused more conflicting phylogenetic signals for ST2 (Fig. 3).

**Fig. 4:**
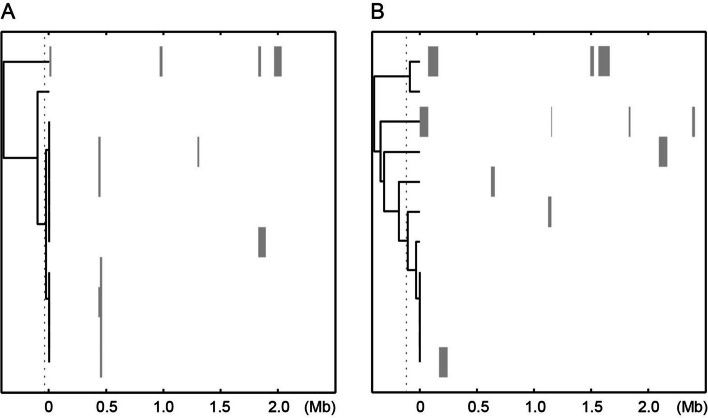
Visualization of the BRATNextGen results identifying recombination events. Eleven ST2 genome sequences are shown in panel A. Eleven ST215 genome sequences are shown in panel B. The left area in the panels is the proportion of shared ancestry (PSA) tree illustrating groups of sequences with recombination events in common. The hatched line shows the cutoff level selected. The right area in the panels shows the recombination events, which are indicated by gray horizontal bars along the genomic nucleotide positions indicated on the X-axis. Recombination events with overlapping nucleotide positions in multiple genome sequences indicate a common origin

To further investigate recombination events in the genomes of the ST2 and ST215 isolates, we used BratNextGen software and detected recombination events, which are graphically displayed in Fig. 3. There were nine recombination events in the ST2 genome analysis, with an average size of 30 544 bp, two of which partially overlapped, suggesting recombination hotspots in this region (Fig. 4). According to the analysis of the ST215 genomes, 11 distinct events were detected, with an average size of 46,245 bp and no genomic location overlap.

We identified antibiotic resistance and virulence genes in the recombining nucleotide stretches and in parts of the genome with no recombination signal by BRATNextGen analysis (Supplementary Table S4). Antibiotic resistance genes (ARGs) found in the recombining genomic regions included *bacA*, *norA*, *evgA, gyrA*, *msbA*, *glpT*, and *tetR,* and the virulence genes in these regions included *capA, capB, capC, capD, sspB, gehD, aae,* and *sspA* (Supplementary Table S5). Among the ST2 genomes, ARGs were more common in the recombinant regions than expected by chance (*p* = 0.008), while virulence genes were not significantly more common. In contrast, among the ST215 genomes, virulence genes were more common in the predicted recombinant regions than they were by chance (*p* = 0.021), while ARGs were not significantly more common in these regions. Alignments of the ST2 and ST215 genomes have been posted as open science data in the European general-purpose open-access research data repository Zenodo hosted by CERN [26].

### Mapping of genes encoding antimicrobial resistance and virulence

The presence or absence of genes described as ARGs or virulence genes were mapped in short-read genome data of the 11 ST215 isolates and 11 ST2 isolates (Supplementary Table S1). We identified 30 ARGs, 26 of which were assigned to specific antimicrobial agent classes and 35 of which were putative virulence genes. Fifty of these genes, 22 ARGs (*mecA, mecR1, blaZ, norA, dfrC, bacA, ileS, blal, murA, carA, fabl, parC, evgA, mgrA, msbA, rpsL, gyrA, srmB, glpT, tetR, ykkC*, and *aac(6´)-le-aph(2´´)-la*) and 29 virulence genes (*sitA, sitB, sitC, graR, graS*, SE0415, SERP0296, *geh1-*Lipase, *sepA, aae, atle, embp, ebp, capA, capB, capC, capD, dltA, dltB, dltC, dltD, mprF, sspB, gehC, gehD, sdrF, sdrG, sdrH,* and *sspA*), were present among all the ST2 and ST215 genomes (Supplementary Table S5). Among the genes that differed in presence among any pair of isolates, some patterns were related to a specific genetic background (ST), time, or place, as outlined in Table 1. The *fusB* gene encoding fusidic acid resistance was present in all the isolates except for the ST2 isolates from Umeå and Östersund in Sweden (the SE07-SE11 isolates). The gene *tetK,* encoding resistance to the tetracycline class of antibiotics, appeared to have been lost over time in the ST215 isolates and was not present in any of the ST2 isolates. The virulence genes *aap, icaA, icaB, icaC icaD* and *icaR* were present in all the ST2 isolates but were consistently absent among the ST215 isolates (Table 1). In the completed genome of the ST215 isolate SE14, the antimicrobial resistance gene *ermC* was found to be located on the plasmid, while the other resistance genes of SE14 were on the chromosome. None of the putative virulence genes in the SE14 genome were on the plasmid.

**Table 1:** Antibiotic resistance and virulence genes differing between 22 *S. epidermidis* genomes of ST2 or ST215

Isolate ID	Sequence type		Presence ( +) or absence (-) of gene													
			Antibiotic resistance genes								Virulence genes					
		*msrA*	*qacA*	*qacB*	*fusB*	*ermC*	*mupA*	*tetK*	*cat*		*aap*	*icaA*	*icaB*	*icaC*	*icaD*	*icaR*
SE01	ST2	+	+	+	+	-	+	-	-		+	+	+	+	+	+
SE02	ST2	+	+	+	+	-	+	-	-		+	+	+	+	+	+
SE03	ST2	+	+	+	+	-	-	-	-		+	+	+	+	+	+
SE04	ST2	+	+	+	+	-	-	-	-		+	+	-	+	+	+
SE05	ST2	+	+	+	+	-	+	-	-		+	+	+	+	+	+
SE06	ST2	+	+	+	+	-	-	-	-		+	+	+	+	+	+
SE07	ST2	-	+	+	-	-	-	-	-		+	+	+	+	+	+
SE08	ST2	-	+	+	-	+	-	-	-		+	+	+	+	+	+
SE09	ST2	-	+	+	-	+	-	-	-		+	+	+	+	+	+
SE10	ST2	-	+	+	-	-	-	-	-		+	+	+	+	+	+
SE11	ST2	-	+	+	-	-	-	-	-		+	+	+	+	+	+
SE12	ST215	-	+	+	+	-	-	-	-		-	-	-	-	-	-
SE13	ST215	-	-	-	+	-	-	-	-		-	-	-	-	-	-
SE14	ST215	-	+	+	+	+	-	-	-		-	-	-	-	-	-
SE16	ST215	-	+	+	+	-	-	+	-		-	-	-	-	-	-
SE17	ST215	-	-	-	+	-	-	+	-		-	-	-	-	-	-
SE18	ST215	-	+	+	+	-	-	+	-		-	-	-	-	-	-
SE19	ST215	-	-	-	+	-	-	+	-		-	-	-	-	-	-
SE20	ST215	-	-	-	+	-	-	+	-		-	-	-	-	-	-
SE21	ST215	-	-	-	+	-	-	+	-		-	-	-	-	-	-
SE22	ST215	-	+	+	+	-	-	+	-		-	-	-	-	-	-
SE23	ST215	-	-	-	+	-	-	+	+		-	-	-	-	-	-

**Fig. 5:**
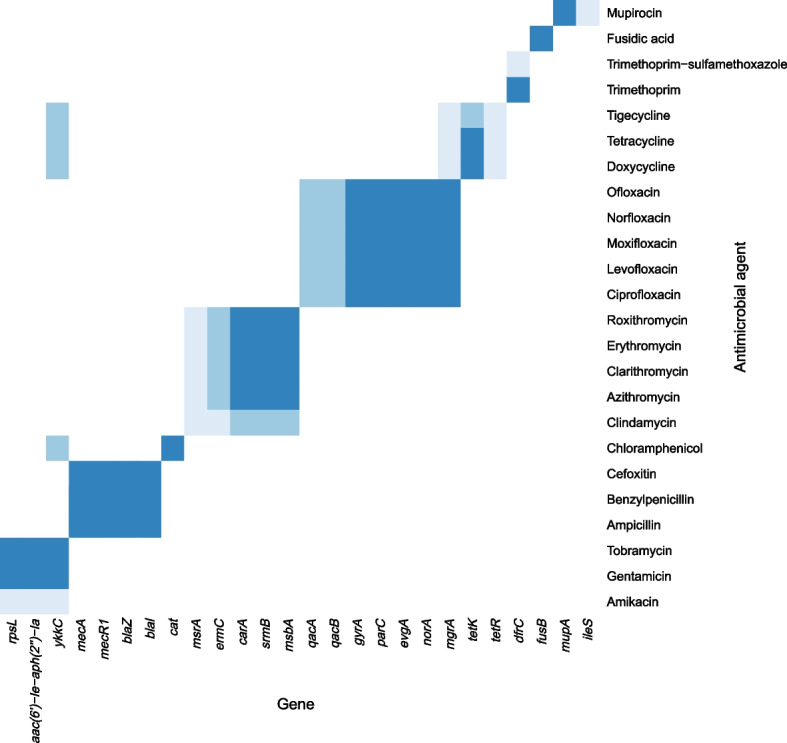
Color gradient visualization of agreement between antimicrobial resistance predicted by genome data and phenotypical resistance as determined by susceptibility analysis of 22 *S. epidermidis* ST2 and ST215 isolates. Dark blue represents > 90% agreement, medium blue represents 50—90% agreement, and light blue represents < 50% agreement. The Resistance Gene Identifier version 4.2.2 was used for resistance prediction

To investigate the agreement between antibiotic ARGs in the genome and phenotypic antimicrobial resistance, the presence or absence of a gene (or presence/absence of a resistance mutation in *gyrA*, *parC*, or *rpsL*) was matched with the antimicrobial susceptibility testing results. In general, we found good agreement by analyzing 26 genes that we could assign to a specific antimicrobial agent class (Fig. 5).

### SNP accumulation over time among the ST215 genomes

After removing the recombined nucleotide stretches, we found a correlation between the clinical sampling date and the accumulation of nucleotide substitutions in the ST215 core genomes isolated from 1995 to 2008 by applying a Monte Carlo-based test and by a Mantel test (*P* = 0.042 and *P* = 0.01, respectively) (Supplementary Fig. S1). In contrast, testing of the ST2 genomes indicated no time dependence.

## Discussion

In this study we completed a nosocomial multidrug-resistant isolate genome of *S. epidermidis* ST215 and performed comparative analyses with 10 additional ST215 draft genomes and phenotypically similar nosocomial ST2 draft genomes. Our findings of evolutionary processes in the genome of ST215 and of antibiotic susceptibility in the ST215 and ST2 isolates fit a scenario of independent emergence of multidrug-resistant *S. epidermidis* from diverse genetic backgrounds within the *S. epidermidis* population [22, 27]. However, whether ST215, like ST2, will expand geographically and become a nosocomial pathogen worldwide is unclear.

The findings illustrate the need for continuous surveillance of multidrug-resistant *S. epidermidis* in healthcare and the importance of antimicrobial stewardship to ensure optimal clinical patient management. Knowing that ST215 and other nosocomial lineages of *S. epidermidis* harbors extensive antibiotic resistance and virulence genes highlights the need for novel antimicrobial therapies. Rapid identification of these nosocomial lineages can enable tailored therapies, ultimately improving patient outcomes. The overrepresentation of virulence genes in ST215 suggests a higher potential for causing infections, indicating the need for enhanced infection control measures to prevent transmission among patients.

In 2018, the ECDC published a rapid risk assessment stating that several endemic multidrug-resistant *S. epidermidis* strains predominate across healthcare systems globally [28]. According to the ECDC, increasing resistance of *S. epidermidis* to multiple antimicrobial agents that are considered first-line agents is a concerning trend that may limit treatment options for indwelling and prosthetic device infections that are difficult to treat [28]. As early as 2005, our research group published the in-hospital persistence of two antimicrobial-resistant *S. epidermidis* clones at the clinics of thoracic surgery, orthopedic surgery, and infectious diseases and at the intensive care unit [29]. One of these was the worldwide disseminated clone named ST2, which was originally described as internationally disseminated a few years earlier [10, 14], while the other clone was a newer clone, ST215, which, to our knowledge, has been disseminated only among hospitals in Northern Europe [11, 21, 22, 30]. The comparative work presented here on the ST2 and ST215 lineages shows that both lineages are resistant to multiple antimicrobial agents, including agents that are cornerstones in the treatment of skin, soft tissue, and bone infections, severely limiting treatment options for these infections [11, 17]. The two lineages have distinct phylogenetic origins but are strikingly similar in terms of ARGs. The antimicrobial gene content varied between lineages or individual isolates for only a handful of genomes, suggesting that ST2 and ST215 are suitable for hospital environments with high antimicrobial pressure and have the capacity to incorporate additional genes when necessary. We note that the ST2 isolates analyzed here were susceptible to vancomycin, linezolid and rifampicin, which contrasts with other described near-pandrug-resistant ST2 isolates [18]. As was shown for *S. epidermidis* isolates from France and Denmark, different treatment traditions and resistance situations between countries may explain the antimicrobial resistant gene content in *S. epidermidis* (P0052 ECCMID 2023). We found high concordance between the antimicrobial resistance phenotypes and ARGs identified in the genome of an isolate, suggesting that genome information could be used to predict phenotypic resistance in *S. epidermidis,* a finding that agrees with the results of more extensive investigations of this topic in, e.g., *Staphylococcus aureus* [31].

We detected multiple recombination nucleotide stretches with an average size of 30–50 kb that contained genes encoding antimicrobial resistance or putative virulence factors. In accordance with recent proposals from other researchers, our findings of overrepresentation of these genes in recombined regions suggested that recombination is a vital mechanism for hospital adaptation [27]. In the case of ST215, the virulence genes were overrepresented in recombined regions, suggesting recent imports of virulence genes into the core genome of ST215. One interpretation of these findings is that selective pressures in the hospital environment not only demand antimicrobial resistance but also require virulence genes, perhaps increasing the capacity to colonize the skin of patients and infect implanted medical devices. We note that ST215 lacks the *icaADBC* operon and the *aap* gene, corroborating previous reports that these genes are absent in ST215 and suggesting that other genes are involved in the process of biofilm formation by ST215 isolates [21, 22]. ARGs were not overrepresented in the recombining genomic regions of ST215, indicating that in this set of isolates from Sweden, most of the ARGs, possibly excluding *ermC*, which was on the sequenced plasmid of isolate SE14, are part of the core genome. In other words, the parts of the genomes encoding ARGs among the ST215 isolates do not display a conflicting phylogenetic signal in relation to the core genome. The results of the ST2 analysis suggested a more complex history of antibiotic resistance gene acquisition; we found overrepresentation of resistance genes but not virulence genes in the recombining genomic regions. It thus seems that in the ST2 genomes from Sweden and Australia, virulence genes are integral parts of the core genome, while recombination events at multiple times have contributed to altering the antimicrobial resistance phenotypes of the bacterium, in line with recent findings that these genes are associated with mobile parts of the genome that help the bacterium adapt to environmental factors [27]. The overall strong influence of recombination on the evolution of *S. epidermidis* agrees with comparative work on multiple genomes of *S. aureus* and *S. epidermidis*, where recombination was found to be much more common in *S. epidermidis*, in which 40.0% of the genes were impacted, than in *S. aureus*, in which 21.2% of the genes were impacted [24]. Additionally, confirming previous findings, we found that staphylococcal cassette chromosome *mec* elements were often adjacent to recombining regions [14, 24, 30, 32].

According to the scientific literature, the ST2 lineage was present worldwide in 1996–1999, with isolates in Argentina, Bulgaria, Columbia, Cape Verde, Denmark, Greece, Iceland, Hungary, Italy, Japan, Mexico, Portugal, and Uruguay [14, 30]. A study including older blood culture isolates from the Swedish hematology unit 1980–2009 showed that ST2 isolates were common in Sweden in the 1980s but that only two ST215 isolates were detected before 1990 [20]. The global geographical dissemination of ST2 at early time points strongly suggests that this lineage emerged long before ST215. Assuming that ST2 is older but that other properties regarding the clinical importance of the two lineages are similar, as indicated by this work, ST215 is a novel threat that may follow the ST2 example of global dissemination. Alternatively, the ST2 lineage is inherently more effective at dissemination than the ST215 lineage is, and its absence in most parts of the world is the result of underreporting. This explanation, however, seems less likely given the experience from Sweden, where the two lineages appear at similar frequencies, causing healthcare-associated infections mainly in orthopedics and thoracic surgery [11, 21, 29]. A third possibility that we cannot rule out but regard as more unlikely is that different antimicrobial pressures and/or differences in hygiene measures have forced ST2 strains to disseminate globally more rapidly, while these pressures and measures have kept ST215 strains at bay. However, our own local experience suggests that hygiene measures and antibiotic stewardship efforts were equally unsuccessful at eradicating ST2 and ST215. Finally, the very recent emergence of ST215 is supported by the fact that we could detect a temporal signal in the SNP accumulation in ST215 after removal of recombined regions. Temporal signals are easily obliterated by high rates of recombination because each recombined stretch of nucleotides may contain many SNPs that, with evolutionary time, overwrite temporal signals created by a clock, such as SNP accumulation [33].

This study has several limitations, including the small sample size of *S. epidermidis* isolates and the fact that we have not studied other clinically important healthcare-associated genetic lineages of *S. epidermidis*, in addition to the ST2 and ST215 lineages. Another limitation is that only one isolate per infection was selected for sequencing, leading to population diversity within an infection site not being accounted for [27, 34]. More comprehensive studies of healthcare-associated *S. epidermidis* are needed to further clarify the most important drivers of the worrisome trend of increasing antimicrobial resistance in *S. epidermidis*.

In conclusion, we studied two distinct phylogenetic lineages of *S. epidermidis* that share an ecological niche in hospitals where they colonize healthcare workers, the hospital environment and patients [7, 35]. We found that the two lineages have similarly evolved and acquired antibiotic resistance and virulence genes through large recombination events. The evolution of the nosocomial ST215 lineage exemplifies that there is a thin line between commensal bacteria and successful pathogens.

## Materials and methods

### Strains

We used clinical *S. epidermidis* isolates cultured from human specimens in Perth, Australia, and in Umeå and Östersund, Sweden. We selected these *S. epidermidis* strains to represent two previously described highly antibiotic-resistant hospital genetic lineages distributed between multiple hospitals. The isolates were previously characterized using pulsed-field electrophoresis and spanned a relatively long period between 1995 and 2008. Twenty-three isolates (12 from PFGE type A1 and 11 from PFGE type D) were included (Supplementary Table S1).

### Antibiotic susceptibility testing

We identified the clinical *S. epidermidis* isolates at the species level by matrix-assisted laser desorption/ionization time-of-flight mass spectrometry (MALDI-TOF MS) and the Biotyper 2.0 database (Bruker Daltonics, Bremen, Germany) [36]. The isolates were analyzed for antibiotic susceptibility with MIC determination for 31 antimicrobial agents according to the European Committee on Antimicrobial Susceptibility Testing (EUCAST) (v. 13.0, www.eucast.org). The tests were performed at the EUCAST Development Laboratory in Växjö, Sweden.

### Genome sequencing and assembly

The *S. epidermidis* isolates were subjected to whole-genome sequencing on the Illumina HiSeq 2000 platform (SciLifeLab, Uppsala, Sweden). DNA preparation, library construction, and genome sequencing were performed according to the manufacturer’s instructions. We used the ABySS 2.0 de novo assembler for short-read sequence data [37]. To determine the STs of the isolates, we analyzed single nucleotide polymorphisms (SNPs) using the *S. epidermidis* MLST 1.8 pipeline available at the Center for Genomic Epidemiology with default settings and whole-genome sequence scaffolds (http://www.genomicepidemiology.org/) [38].

### Finishing the genome of isolate SE14

The SE14 isolate was in addition sequenced using MinION (Oxford Nanopore Technologies, UK; flow cell R 9.4.1, LSK-SQK109). All the sequenced SE14 reads were assembled using Unicycler (v0.4.8; Wick, R. R. et al. (2017)) software. Reads that did not map to any of the assembled contigs were assembled using metaspades (v3.14.0 Bankevich, A. et al. (2012)) and canu (v2.0 Koren, S. et al. (2017)) but were not supported by read coverage.

### Multiple sequence alignment and phylogenetic analyses

The 23 de novo assembled *S. epidermidis* genomes and 60 public *S. epidermidis* genomes were analyzed (Supplementary Table S3). We used a stepwise alignment strategy and created multiple alignments in groups of ten genomes using progressiveMauve v12 [39] with the *S. epidermidis* ATCC 12228 genome as the index. The groups were merged into a single alignment using the indexed nucleotide positions. We estimated genetic distances between genomes using the Kimura 2-parameter distance before applying the neighbor-joining algorithm to construct a neighbor-joining phylogenetic tree with the standard settings in MEGA 5.13 (http://www.megasoftware.net/) [40]. We constructed a clade wise phylogenetic network using the neighbor-net algorithm in the SplitsTree3.2 package, which was designed to account for gene genesis, gene loss, horizontal gene transfer, or recombination [41].

### Phylogeny

The de novo assembled genome of SE14, as well as 1,361 public genomes of the species *S. epidermidis* according to gtdb r207 [42], was aligned in a pairwise manner against the genome of the species type strain NBRC 100911 (GCF_006742205.1) using progressiveMauve (v.2015_02_13) [39]. Each alignment was arranged according to the reference coordinates using the python script x2fa.py included in CanSNPer v.1.0.8 (10.1093/bioinformatics/btu113) and concatenated to a multi-FASTA alignment. The phylogeny was calculated with IQ-TREE v 2.2.0.3 with automatic model finding (-m TEST), resulting in the evolutionary model GTR + F + I + G according to the Bayesian Information Criterion (BIC) [43]. iTOL v6.8 was used to visualize and export the phylogenetic data (10.1093/nar/gkab301). All software except iTOL was executed using the workflow manager Snakemake (v.6.2.1) [44] and installed using conda (v4.8.5) with Bioconda [45] and conda-forge channels.

### Detection of antibiotic resistance and virulence genes

We searched for ARGs in the whole-genome read data of 23 *S. epidermidis* isolates generated in this work using the software RGI, Resistance Gene Identifier version 4.2.2 within the search engine Card version 3.0.0 of the Comprehensive Antibiotic Resistance Database (Card) [46]. We applied the discovery criterion “*predict perfect, strict and loose hits”* and the sequence quality setting *“high quality reads”* to predict the content of resistance genes in each genome from the nucleotide data. The resulting hits with a *% identity of matching region* greater than 30 and *% Length of Reference sequence* greater than 50 were included in our analysis. For each detected antibiotic resistance gene among the reads of a specific isolate, the presence of that gene was manually checked among the reads for all other isolates to avoid misclassification of the resistomes resulting from a potential difference in sequence quality among the isolates. This was performed using the software BLAST + 2.6.0, with the MegaBlast algorithm for highly similar sequences (NCBI, the National Center for Biotechnology Information, USA) using a threshold of 90% nucleotide identity over 90% of the target sequence length. If the sequence length was less than 100%, frame-shift mutations were searched for, and the sequences predicted as nonfunctional genes were discarded. Preferably, a Card database sequence of *S. epidermidis* was used, and if lacking in the database, *S. epidermidis* sequences corresponding to the hit sequence in Card were identified in other public databases, such as NCBI or UniProt [47]. If the resistance gene was not found in a public *S. epidermidis* sequence, the sequence of the most closely related taxon was used (Supplementary Table S5). Finally, *S. epidermidis* genes described as virulence genes were collected from the scientific literature as reviewed by Otto and from the Card database [48, 49]. The sequences of all these genes were retrieved and used as target sequences in searches of the genome data of the 23 *S. epidermidis* isolates. For the ARGs, the criterion of at least 90% nucleotide identity over 90% of the target sequence length was used for BLAST searches. Notably, all the genes identified among the 23 isolates (Supplementary Table S5) were highly similar to a target sequence with at least 96% nucleotide identity over 99–100% of the sequence length.

### Recombination analysis

To detect and locate recombining events and to obtain recombination-free input sequences for analyses of SNP accumulation over time, we applied BRATNextGen software, which is based on Bayesian statistical models [50]. The ST2 and ST215 genomes were analyzed separately. The proportion of shared ancestry (PSA) tree cutoffs was set at three clusters for ST2 and five clusters for ST215, supported by a clear clustering pattern in the PSA tree. We used 1000 iterations within the hidden Markov model and 100 permutations, and the significance level was set to 5%. The genomic regions predicted as recombination events as well as the entire genome were analyzed for the content of virulence genes and ARGs using the software BLAST + 2.6.0 (NCBI, USA) with the threshold of at least 97% nucleotide identity over at least 40% of the sequence length. These data were used for testing the overrepresentation of antibiotic resistance and virulence genes in recombining regions as described below.

### Accession numbers

The raw sequence data and assembled genome sequences of all the isolates were deposited in the NCBI database under the BioProject accession number PRJNA557130 [51].

### Supplementary Information

Supplementary Material 1.

## Data Availability

The sequence data supporting the conclusions of this article are available in the NCBI database under the BioProject accession number PRJNA557130 and in the European general-purpose open-access research data repository Zenodo hosted by CERN [26, 51].

## Abbreviations

EUCAST: the European Committee on Antimicrobial Susceptibility Testing
MLST: Multilocus sequence typing
PSA: proportion of shared ancestry
SNP: single nucleotide polymorphism
ST: sequence type
