# Supplementary material for: Completed genome and emergence scenario of the multidrug-resistant nosocomial pathogen Staphylococcus epidermidis ST215
Source: BMC Microbiol. 2024 Jun 19;24:215. doi: 10.1186/s12866-024-03367-5 (PMC11186124; doi:10.1186/s12866-024-03367-5)
Supplement: Supplementary file 1 — Supplementary Material 1. [file 12866_2024_3367_MOESM1_ESM.zip › Supplementary Fig S1.pdf]

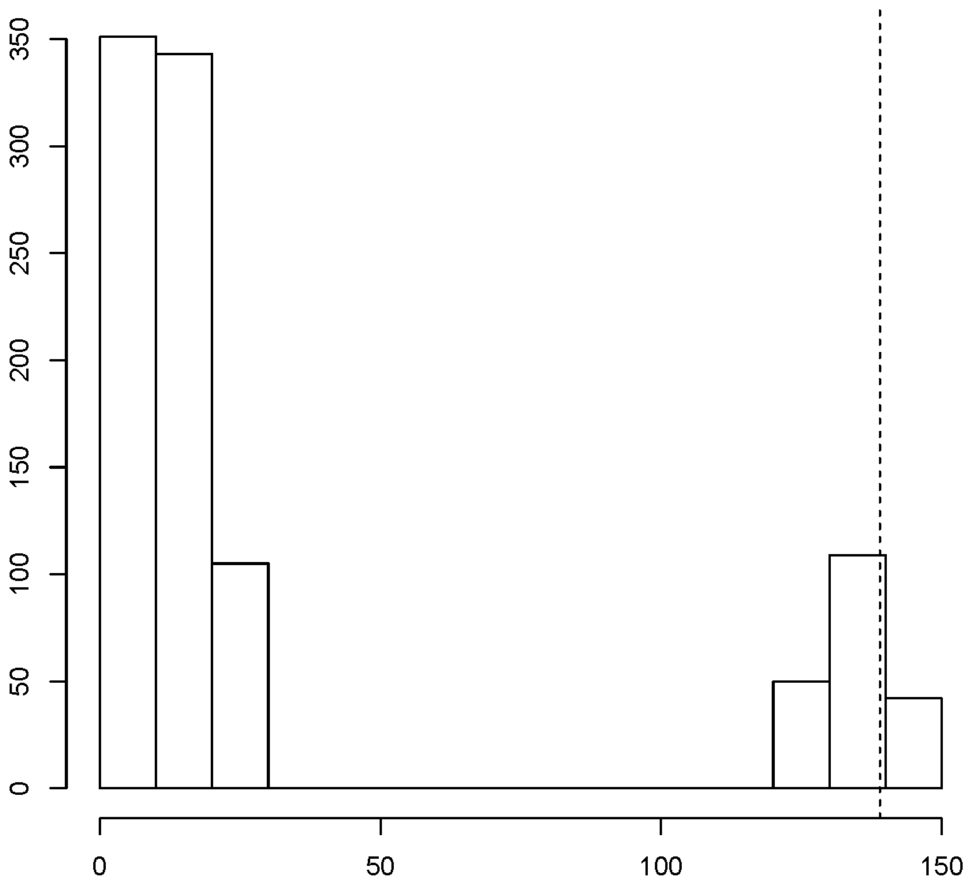

Supplementary Fig. S1. Distribution of the test statistics obtained in the Monte Carlo simulations as described in section “Statistical test for time dependence of SNP accumulation”, for the 11 ST215 isolates. The vertical dashed line indicates the test statistic δ observed from the empirical data ($\delta = 139$, P = 0.042).
