# Supplementary material for: Completed genome and emergence scenario of the multidrug-resistant nosocomial pathogen Staphylococcus epidermidis ST215
Source: BMC Microbiol. 2024 Jun 19;24:215. doi: 10.1186/s12866-024-03367-5 (PMC11186124; doi:10.1186/s12866-024-03367-5)
Supplement: Supplementary file 1 — Supplementary Material 1. [file 12866_2024_3367_MOESM1_ESM.zip › Supplementary File 1.docx]

**Supplementary File 1**

**Statistical test for the time dependence of SNP accumulation.**

To investigate whether patients were infected by strains that locally evolved from a recent common ancestor, or were infected by independent strains, a test was developed to investigate time-dependence between isolates. The null hypothesis was that patients were infected by independent strains, and the alternative hypothesis was that at least two strains shared a recent common ancestor. A SNP should agree with time; hence, backward and additional mutations at the same genomic position are not allowed. Our test statistic was the number of SNPs in agreement with time among the tested isolates.

The ST215 isolates, sampled from Umeå University Hospital, were ordered with respect to when the samples were taken, so that the oldest and most recent isolates were indexed 1 and 11, respectively.

The sequences were aligned, and only bases with observations from all the isolates were included. The aligned sequences, including m bases, were denoted

where

*j*=*1,…,n*. Here the base were either A, C, G or T. Let denote the observations from the *i*th base, *i.e.*

*i*=*1,…,m*. A sequence is said to be *changed and in agreement* if it contains exactly one change, *i.e.* if the indicator variable, where

*i*=*1,…,m*. Let the test statistic denote the total number of bases that was changed and in agreement, *i.e.*

Under the above assumptions the mean value of the test-statistic will increase with the number of patients infected at the hospital.

Monte Carlo simulations were used to estimate the distribution of under the null hypothesis. The procedure involved three steps:

1) *n* individuals, denoted were drawn without replacement from individuals *1,…,n*. Resulting in the ordered pseudo dataset

2) The simulated replicate was calculated based on the data described above.

3) Steps 1 and 2 were repeated B times (B=1000) resulting in the replicates

and the test’s p-value was estimated as

,

Prior to the test, all recombining regions that were detected by BratNextGen were removed. As a complementary test, Mantel’s test was used to evaluate whether there was a significant correlation between pairwise genetic Hamming distances and time-differences between genomes within each of the genetic groups ST2 and ST215 (the number of days between clinical sampling was used as the time-difference variable) (1).

**Statistical test for overrepresentation of antibiotic resistance and virulence genes in recombined regions**

The null hypothesis was that the virulence and antibiotic resistance genes were equally distributed throughout the genomes. The alternative hypothesis was that the virulence or antibiotic resistance genes were overrepresented in the recombined areas. The population of virulence genes tested was the same as those discovered by Otto et al. (2), and the tested antibiotic resistance genes the same were the same as those discovered by using RGI software in CARD (3).

The length of the recombined areas was identified as described in “Recombination analysis”, where is the sum of all recombined genomic regions in all isolates of ST2 or ST215, respectively. is the remaining length of the genomes, i.e. + is the total length of all the genomes. is the number of genes, either virulence or antibiotic resistance genes, found in and is the number of genes found in . Note that is the total number of genes identified in the considered isolates. Given that the null hypothesis is true, the expected number of genes in the recombined areas is given by

and the expected number of genes in the rest of the genomes were given by

To test this hypothesis a chi-square test was used with the test statistic

,

Where is chi-square distribution with one degree of freedom.

**Statistical test for difference in genetic diversity between genetic groups**

Hypothesizing that the ST215 isolates collected at Umeå University Hospital in 2003-2008 evolved locally and more recently while the ST2 strains, which have been reported worldwide were more ancient, we tested the difference in genetic diversity between these genetic groups. Due to the dependency structure in the data, two-sample tests for comparing intra-individual genetic sequence diversity between populations (4, 5) were used. All the statistical tests were evaluated at the 5% significance level.

References

1. Diniz-Filho JA, Soares TN, Lima JS, Dobrovolski R, Landeiro VL, de Campos Telles MP, et al. Mantel test in population genetics. Genet Mol Biol. 2013;36(4):475-85.

2. Otto M. Molecular basis of Staphylococcus epidermidis infections. Semin Immunopathol. 2012;34(2):201-14.

3. Jia B, Raphenya AR, Alcock B, Waglechner N, Guo P, Tsang KK, et al. CARD 2017: expansion and model-centric curation of the comprehensive antibiotic resistance database. Nucleic Acids Res. 2017;45(D1):D566-D73.

4. Gilbert PB, Rossini AJ, Shankarappa R. Two-sample tests for comparing intra-individual genetic sequence diversity between populations. Biometrics. 2005;61(1):106-17.

5. Giorgi EE, Bhattacharya T. A note on two-sample tests for comparing intra-individual genetic sequence diversity between populations. Biometrics. 2012;68(4):1323-6; author reply 6.
